# Effects of natural reduced water on cognitive functions in older adults: A RCT study

**DOI:** 10.1016/j.heliyon.2024.e38505

**Published:** 2024-09-28

**Authors:** Takamitsu Shinada, Keisuke Kokubun, Yuji Takano, Hikari Iki, Koki Kobayashi, Takeki Hamasaki, Yasuyuki Taki

**Affiliations:** a Smart-Aging Research Center, Tohoku University, Sendai, 980-8575, Japan; b Graduate School of Management, Kyoto University, Kyoto, 606-8501, Japan; c Department of Psychology, University of Human Environments, Matsuyama, 790-0825, Japan; d Department of Aging Research and Geriatric Medicine, Institute of Development, Aging and Cancer, Tohoku University, Sendai, 980-8575, Japan; e Laboratory of Functional Water, Food and Energy, Department of Bioscience and Biotechnology, Faculty of Agriculture, Kyushu University, Fukuoka, 812-8581, Japan

## Abstract

Oxidative stress and diabetes increase the risk of cognitive decline and dementia. Natural reduced water contains active hydrogen (hydrogen radicals), eliminates reactive oxygen species, and has antidiabetic effects. However, whether natural reduced water affects human cognitive function is unknown. Therefore, we implemented a double-blind intervention experiment in which participants consumed 1 L of natural reduced water or tap water daily for 6 months. The participants were healthy older adults living in Japan. The intervention group showed significant improvements in cognitive functions of attention function (p < 0.01) and short-term memory (p < 0.05). These results indicate that the continuous intake of natural reduced water improves several cognitive functions.

## 1 Introduction

Natural reduced water contains active hydrogen (hydrogen radicals or hydrogen atoms), which reduces oxidation. It is rare worldwide and is found in Germany (Nordenau Water) and Mexico (Tracote Water). In Japan, natural reduced water is collected deep underground in Hita City, Oita Prefecture, and marketed as Hita Tenryosui water. Hita Tenryosui water and Nordenau water, which are natural reduced waters, have antioxidant and antidiabetic effects [[1], [2], [3], [4]]. Oxidative stress is associated with the development of cancer, diabetes, neurodegenerative, cardiovascular, respiratory, and immune disorders. Furthermore, overproduction of reactive oxygen species (ROS) can cause diseases [[5], [6], [7], [8]]. The brain is more susceptible to ROS because it has a high oxygen demand and is rich in polyunsaturated fatty acids [9]. In regions of the brain rich in amyloid-β, a symptom of Alzheimer's disease, protein and lipid oxidation has been observed [10].

The relationship between hydrogen and cognitive function has been examined in previous studies [11]. In mice, the consumption of hydrogen water reduces oxidative damage and memory impairment [12]. Molecular hydrogen acts as an antioxidant, removing ROS and mitigating oxidative damage [13]. Cognitive decline has also been observed in response to oxidative stress [14]. Active hydrogen (hydrogen radicals and hydrogen atoms) in natural reduced water is known to be active agents with reduced energy [15,16]. Hydrogen radicals have a stronger reducing effect than molecular hydrogen radicals [17,18]. In natural reduced water, hydrogen radicals bind to minerals to create mineral nanoparticle hydrides that maintain reduced energy [19]. Although it is known that water containing molecular hydrogen improves cognitive function in animal studies, the effect of natural reduced water containing hydrogen radicals on cognitive function in humans is not known. Therefore, our main objective was to investigate the effects of continuous consumption of natural reduced water on cognitive function in humans.

An anti-obesity effect was also observed in another study after 6 months of natural reduced water intake [20]. In human experiments, plasma triglycerides and total cholesterol levels related to obesity improved by 2 months of intervention of natural reduced water consumption [1]. Animal cell experiments have shown that natural reduced water can activate aquaporins [21]. Aquaporins mediate water homeostasis in the body [22,23], which passes through water molecules. Therefore, natural reduced water has the potential to improve human body structure, including body fat percentage and body water content. However, previous studies have suggested that natural reduced water indirectly affects psychological functions. In animal experiments with rats, natural reduced water was effective in reducing conditioned fear stress and anxiety in the conditioned fear test and the elevated cross maze test [24]. Previous human studies have shown that reduced natural water activates natural killer (NK) cells [21], and surgical stress reduces NK cells [25]. Stress is also correlated with subjective well-being [26].

Previous studies on natural reduced water have focused mainly on cells and animals, and there have been few studies on humans, especially older adults. Therefore, this study aimed to determine the effects of natural reduced water intake on cognitive ability in older adults.

## 2 Methods

### 2.1 Study design

**Fig. 1:**
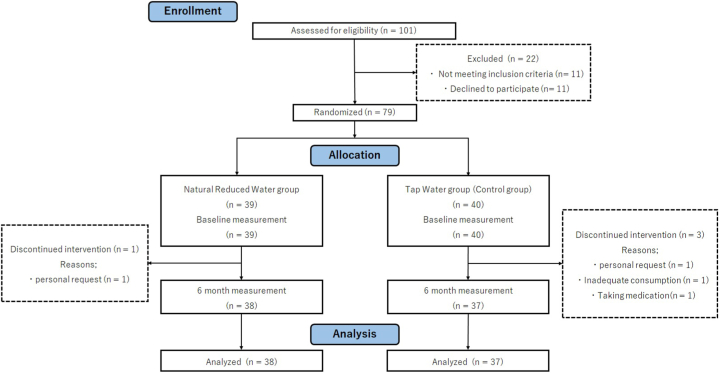
Study flow diagram.

We recruited participants for this experiment through advertisements in local weekly magazines and first screened applicants to ensure they met our inclusion criteria. All participants provided verbal and written informed consent prior to inclusion in the study. After obtaining informed consent, a baseline test was conducted (Fig. 1). Baseline testing included measures of cognitive function, questionnaires on psychological functioning, and measurements of body composition. The testers administered the cognitive function test face-to-face. We used paper-based questionnaires rather than computer-based ones. We divided the participants into two groups using double-blind randomization: one with natural reduced water and the other with tap water. A staff member unrelated to the study randomly assigned the participants to two groups using computer-generated random numbers. Neither the researchers, testers, nor participants were informed of which group the participants belonged to. No stratification factors were used to classify the participants. Participants drank 1 L of water per day. The bottle labels were removed for the double-blind experiment and the participants did not know which water they consumed. They were asked to drink water as it was, although they could drink it warm or cold (they did not drink water in another state, such as coffee or tea). Participants filled in a list to record their daily water consumption for the duration of the study. We also checked every 20 days to determine whether the participants received water delivery. There were four dropouts during the intervention. This was due to the lack of the required consumption rate, condition, or request to stop. After 6 months of intervention, participants were administered a post-test with the same content as the pre-test. Since both the intervention and control groups received the same test, any learning effect would not have been a major issue, even if it existed. We provided natural reduced water to the tap water group of participants who had completed the intervention and wanted to drink natural reduced water. The Ethics Committee of Tohoku University Graduate School of Medicine approved the ethical appropriateness of the study (2020-1-631). The study was registered in the UMIN Clinical Trials Registry (UMIN000041945) on September 30, 2020. This study followed CONSORT guidelines.

### 2.2 Inclusion criteria

We recruited healthy older adults aged 65−74 years who were independent in their daily lives.

### 2.3 Exclusion criteria

High water intake can also cause electrolyte abnormalities. Therefore, we excluded participants who were at high risk for electrolyte abnormalities. Specifically, exclusion criteria included the following clinical histories: kidney diseases, heart disease, hepatic cirrhosis, inadequate anti-diuretic hormone syndrome, low thyroid function, diabetes mellitus, hyperlipidemia, mental disorders (polydipsia), emesis and diarrhea. We also excluded participants who were taking medications that were likely to cause hyponatremia: antiepileptics, antidepressants, diuretics, antihypertensives, and antiarrhythmic drugs. The other exclusion criteria were severe audiovisual impairment and the consumption of natural reduced water within the past year.

### 2.4 Materials used for intervention

For this study, 2 L × 10 bottles were delivered to the participants’ homes every 20 days with the labels removed from the bottles. Natural reduced water (Hita Tenryosui water) was used, which is commercially available. Hita Tenryo-Sui Co., Ltd. provided natural reduced water and tap water. Tap water was boiled and bottled at Hita Tenryo-Sui Co., Ltd. The water quality of Hita Tenryosui was well controlled. The data comparing the composition of the two types of water used in this study (Hita Tenryosui and tap water) are shown in Table 1. Compared to tap water, Hita Tenryosui water (natural reduced water) contains higher concentrations of minerals such as soluble silicon, sodium, potassium, and hydrogen carbonate ions. The pH is 8.3 (slightly alkaline). Active hydrogen measurement data using the 3,5-dibromo-4-nitrosobenzene sulfonate (DBNBS) method are presented in Table 1.

**Table 1:** Composition of natural reduced water and tap water.

Water composition	Natural reduced water	Tap water
Sodium (Na)∗^a^	23 mg/L	13 mg/L
Calcium (Ca)∗^a^	8.6 mg/L	11 mg/L
Pottasium (K)∗^a^	7.8 mg/L	2.9 mg/L
Magnesium (Mg)∗^a^	1.6 mg/L	3.0 mg/L
Soluble silicon (SiO_2_) ∗^a^	84 mg/L	40 mg/L
Sulfate ion (SO_4_^2−^) ∗^a^	3.6 mg/L	14 mg/L
Hydrogen carbonate ion (HCO^3−^) ∗^a^	79 mg/L	27 mg/L
Active hydrogen (Abs450 nm) ∗^b^	0.2069	0.0487

a Survey by the Miyagi Pollution and Sanitation Inspection Center.

b Measurements are performed based on references [27].

### 2.5 Measurement items

#### 2.5.1 Cognitive function

Mini-Mental State Examination (MMSE) [28]: The MMSE measures global cognitive function. It is a 30-point test with a cut-off point of 23/24. This method was not used in the screening tests. However, it has also been used to measure general cognitive function.

Trail Making Test (TMT) [29]: The TMT measures attention and executive functions. The TMT consists of the TMT-A and TMT-B. In the TMT-A, participants connected the 26 numbers on paper with a pencil line, starting with the smallest number as quickly as possible. In TMT-B, participants connected 25 numbers and letters in alternating order with a line as fast as possible. The time required to connect the lines was measured using a stopwatch.

Digit Span (DS) [30]: The DS is used to measure short-term memory. This is a subtest of the Wechsler Adult Intelligence Scale-III. In the DS-F, the tester presented a number of digits; the participants heard and memorized them and then repeated them in the same order. In the DS-B, participants heard the numbers and then repeated them in reverse order.

Verbal fluency test (VFT) [31]: There are two types of VFT, the first involves word recall from semantic categories and the second involves word recall from acronyms. Participants were asked to recall as many words as possible within 60 s and write them on paper.

We used Japanese versions of MMSE [32], TMT [33], DS [34], and VFT [35].

#### 2.5.2 Psychological function

Profile of Mood States 2 (POMS2) [36]: POMS2 evaluates mood state during a given time frame based on seven scales: [anger-hostility], [confusion-bewilderment], [depression-dejection], [fatigue-fatigue-inertia], [tension-anxiety], [vigor-activity], and [friendliness], and the TMD score, which represents the overall negative mood state.

Perceived Stress Scale (PSS) [37]: The PSS is a questionnaire used in numerous studies and has been shown to be predictive of a wide variety of stress responses and comprehensively assess stress. Then reverse scoring was performed. The participants answered questions about their situation in the past month.

Subjective Happiness Scale (SHS) [38]: This measures the degree to which people subjectively perceive happiness and is composed of four items.

For these tests, we used the Japanese versions of POMS2 [36], PSS [37], and SHS [38].

#### 2.5.3 Body composition

Body composition meter (RD-800; TANITA, Japan): We measured body water and body fat rates, as well as various other parameters such as muscle mass, body mass index, bone mass, and basal metabolism.

### 2.6 Sample size

We used G∗Power 3.1.9.7 (Heinrich-Heine-Universität Düsseldorf., Düsseldorf, Germany) to calculate the sample size. In this software, the effect size d_z_ is defined as the following formula:

$$d_{z}=\frac{\left|\mu_{z}\right|}{\sigma_{z}}=\frac{\left|\mu_{x}-\mu_{y}\right|}{\sqrt{\sigma_{x}^{2}+\sigma_{y}^{2}-2\rho_{\text{xy}}\sigma_{x}\sigma_{y}}}$$

where μ^x^ and μ^y^ denote the sample means, σ_x_ and σ_y_ denote the standard deviation in either sample, and ρ_xy_ denotes the correlation between the two random samples. μ_z_ and σ_z_ are the sample mean and standard deviation of the difference z. Using Cohen's "moderate" levels of significance (α) of 5 %, effect size of 0.5, and power (1-β) of 80 %, the number of participants needed in each group was calculated to be 34 [39]. Assuming a drop rate of 10 %, the study was designed to recruit 76 participants (38 each in the intervention and control groups) over the 6-month intervention period.

### 2.7 Data analysis

To compare initial values by group, an unpaired *t*-test was used to assess whether the intervention and control groups were homogeneous. For pre- and post-intervention comparisons between the two groups, two-way repeated measures analysis of variance (ANOVA) was performed to examine the group-by-time interaction and main effects. A paired *t*-test was conducted to examine within-group effects. Additionally, an unpaired *t*-test was used to compare the two groups after the intervention. The level of significance was set at 5 %. We used SPSS ver.25 (IBM, Armonk, NY, USA) to analyze the data.

## 3 Results

We analyzed the results of 38 and 37 participants in the intervention and control groups, respectively, before and after the intervention. There were no statistically significant differences between the two groups regarding mean age, years of education, or sex. There was also no significant difference in the pre-intervention test values between the two groups (Table 2).

**Table 2:** Initial values by groups.

	Intervention group		Control group		**t-value**	**p-value**
	**Mean**	**SD**	**Mean**	**SD**		
**Age**	68.66	2.72	68.89	3.15	0.34	0.73
**Years of education**	13.39	2.01	14.22	2.19	1.70	0.09
**MMSE-J**	29.03	1.17	28.89	1.15	0.50	0.62
**Body fat rate (%)**	30.41	7.64	29.72	7.27	0.40	0.69
**Body water rate (%)**	47.84	3.84	48.13	4.64	0.30	0.77
**TMT-A (s)**	78.97	19.18	77.30	19.42	0.38	0.71
**TMT-B (s)**	102.82	31.89	101.62	25.44	0.18	0.86
**DS-F**	9.03	2.60	9.14	1.65	0.22	0.83
**DS-B**	6.50	2.48	6.35	1.93	0.29	0.77
**VFT (letter)**	9.16	2.54	9.14	3.01	0.04	0.97
**VFT (category)**	10.18	2.20	9.68	2.73	0.89	0.38
**SHS**	4.89	0.82	5.09	0.75	1.10	0.27
**PSS**	22.71	6.44	22.16	7.11	0.35	0.73
**POMS2**	9.79	14.35	5.59	13.22	1.32	0.19
	**N**	**%**	**n**	**%**	**χ**^**2**^	**p-value**
**Male**	12	46	9	32	0.49	0.61
**Female**	26	54	28	68		

**Fig. 2:**
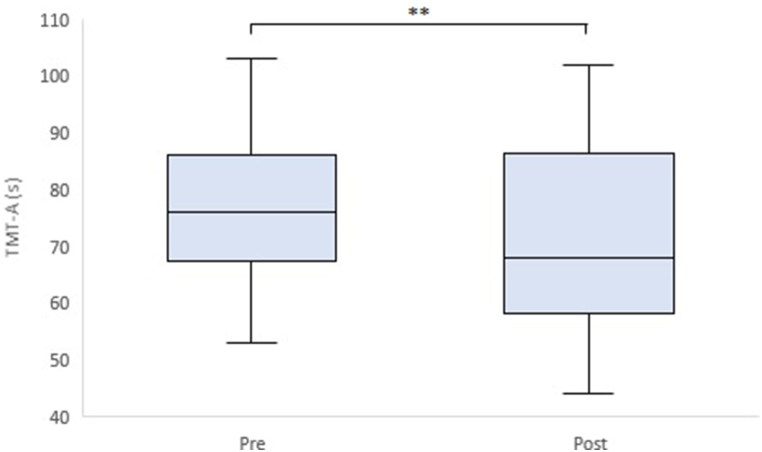
TMT-A box plot (pre-post in the intervention group).

**Fig. 3:**
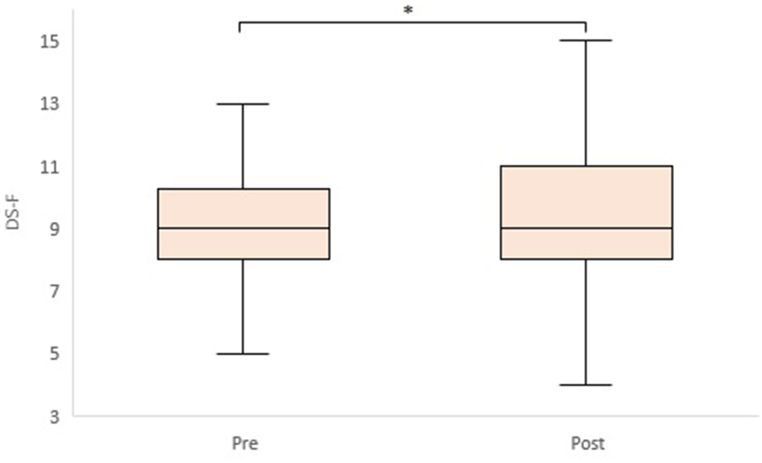
DS-F box plot (pre-post in the intervention group).

We performed a two-way repeated measures ANOVA to examine group-by-time interactions. However, there were no significant group-by-time interactions for any of the items. In contrast, time had a significant effect on body fat rate (F(1,73) = 90.35, p < 0.01), body water rate (F(1,73) = 72.80, p < 0.01), TMT-A (F(1,73) = 6.31, p = 0.01), and DS-B (F(1,73) = 4.96, p = 0.03). Therefore, we performed a paired *t*-test to determine the within-group differences between pre- and post-intervention (Table 3). In the intervention group, the scores of the cognitive function tests, TMT-A (t = 2.82, p < 0.01, d = 0.46), and Digit Span Forward (DS-F) (t = 2.10, p = 0.04, d = 0.34) showed a statistically significant improvement after the intervention (Fig. 2, Fig. 3). Regarding Cohen's effect size guidelines (d = 0.2, small; d = 0.5, medium; d = 0.8, large) [39], both TMT-A and DS-F improved with a small effect size. In DS-B, there was no significant improvement before and after the intervention, but the effect size was larger than in the small group (t = 1.99, p = 0.05, d = 0.32). Other parameters that showed significant improvements within the intervention group included body fat rate (t = 6.30, p < 0.01, d = 1.02) and body water rate (t = 4.99, p < 0.01, d = 0.81). However, these items showed similar improvements in the control group (t = 7.53, p < 0.01, d = 1.23 for body fat rate; t = 8.09, p < 0.01, d = 1.33 for body water rate). Furthermore, the VFT scores improved significantly only in the control group. We also performed an unpaired *t*-test to compare the two groups after the intervention. There were no significant differences between the groups after the intervention (Table 4). However, TMT-A (t = 1.06, p = 0.29, d = 0.25) and DS-B (t = 1.09, p = 0.28, d = 0.25) showed differences between the groups for effect sizes larger than small, indicating a small improvement with intervention.

$$d=\frac{\mu_{1}-\mu_{2}}{\overset{´}{\sigma }}$$

$$\overset{´}{\sigma }=\sqrt{\frac{\sigma_{1}^{2}+\sigma_{2}^{2}}{2}}$$

where μ_1_ and μ_2_ denote the sample means, σ_1_ and σ_2_ denote the standard deviation in either sample.

**Table 3:** Results of the intervention group and the control group.

	Intervention group							Control group						
	Pre		Post		Pre-Post			Pre		Post		Pre-Post		
	Mean	SD	Mean	SD	t-value	p-value	Effect size (d_z_)	Mean	SD	Mean	SD	t-value	p-value	Effect size (d_z_)
**Body fat rate (%)**	30.41	7.64	27.72	7.98	6.30	0.00∗	1.02	29.72	7.27	27.33	7.77	7.53	0.00∗	1.23
**Body water rate (%)**	47.84	3.84	50.29	5.12	4.99	0.00∗	0.81	48.13	4.64	50.70	5.22	8.09	0.00∗	1.33
**MMSE-J**	29.03	1.17	29.16	1.37	0.56	0.58	0.09	28.89	1.15	29.16	0.99	1.43	0.16	0.24
**TMT-A (s)**	78.97	19.18	71.00	16.54	2.82	0.01∗∗	0.46	77.30	19.42	75.49	19.94	0.68	0.50	0.11
**TMT-B (s)**	102.82	31.89	97.87	24.97	1.32	0.19	0.21	101.62	25.44	96.73	24.64	1.25	0.22	0.20
**DS-F**	9.03	2.60	9.50	2.62	2.10	0.04∗	0.34	9.14	1.65	9.19	1.31	0.21	0.84	0.03
**DS-B**	6.50	2.48	7.32	2.77	1.99	0.05	0.32	6.35	1.93	6.70	2.07	1.08	0.29	0.18
**VFT (letter)**	9.16	2.54	9.26	2.54	0.25	0.80	0.04	9.14	3.01	10.57	3.31	2.57	0.01∗	0.42
**VFT (category)**	10.18	2.20	10.53	2.49	1.03	0.31	0.17	9.68	2.73	10.49	2.56	2.56	0.01∗	0.42
**SHS**	4.89	0.82	4.90	0.77	0.15	0.88	0.02	5.09	0.75	5.24	0.79	1.68	0.10	0.28
**PSS**	22.71	6.44	22.13	5.54	0.67	0.51	0.11	22.16	7.11	22.49	6.37	0.31	0.75	0.05
**POMS2**	9.79	14.35	6.87	11.93	1.71	0.09	0.28	5.59	13.22	4.54	14.32	0.64	0.53	0.10

Note: The effect size, d_z_, is calculated using the equation in Section 2.6.

**Table 4:** Between-group comparison of post-intervention results.

	t-value	p-value	Effect size (d)
**Body fat rate**	0.21	0.83	0.05
**Body water rate**	0.35	0.73	0.08
**MMSE-J**	0.02	0.99	0.00
**TMT-A**	1.06	0.29	0.25
**TMT-B**	0.20	0.84	0.05
**DS-F**	0.65	0.52	0.15
**DS-B**	1.09	0.28	0.25
**VFT (letter)**	1.92	0.06	0.44
**VFT (category)**	0.07	0.95	0.02
**SHS**	1.86	0.07	0.43
**PSS**	0.26	0.80	0.06
**POMS2**	0.77	0.45	0.18

Notes: The effect size d is calculated using the following formula.

## 4 Discussion

Natural reduced water is known to have antioxidant, antidiabetic, and other effects. However, the effects of natural reduced water on human cognition, psychological state, and body composition have not been investigated, so we conducted a double-blind study. Consequently, the two cognitive tests showed significant improvements within the intervention group, with small effect sizes.

The first was the TMT-A, which measures the attention function. A previous study indicated that people with diabetes experience a decrease in attentional function [40,41]. High blood glucose levels are known to be associated with cognitive decline [[42], [43], [44], [45]]. Diabetes is a risk factor for the development of Alzheimer's disease (AD) [46], vascular dementia [47], and mild cognitive impairment (MCI) [48,49]. Type 2 diabetes is associated with a 1.5 − 2.5 times higher risk of dementia [42]. Approximately 10 % of people with dementia have diabetes mellitus [50]. The overproduction of factors that contribute to diabetes is the overproduction of ROS [51]. Natural reduced water removes ROS and suppresses the symptoms of diabetes in vitro, in vivo, and in humans [[1], [2], [3]]. In these studies, diabetic patients continuously consumed Hita Tenryosui water for 2 months, and fasting glucose level decreased significantly from 8.14 ± 1.25 nmol/L to 6.99 ± 1.45 nmol/L. Therefore, in this intervention study, we considered natural reduced water with antioxidant and antidiabetic effects to improve attention function.

The second is DS-F, which measures short-term memory. In in vivo experiments, it has been shown that high oxidative stress and ROS production are associated with memory impairment [[52], [53], [54], [55]]. In the human brain, elevated levels of posterior cingulate glutathione (GSH), an antioxidant protein synthesized by the transcription factor Nrf2, are associated with poorer memory function [56]. Hydrogen radicals in natural reduced water is a reducing agent, and vitamin C (ascorbic acid) has antioxidant properties as a reducing agent [57,58]. Vitamin C is rich in the human cerebral cortex, hippocampus, and amygdala [59,60]. Vitamin C supplementation has been shown to decrease hippocampal neuronal cell death in vivo [61], and ROS production is suppressed by vitamin C treatment in vitro [62]. The group without cognitive impairment has been shown to have higher mean vitamin C concentrations than the group of participants with cognitive impairment [63]. Furthermore, among participants without cognitive impairment, blood levels of vitamin C have been shown to be associated with cognitive performance (e.g., short-term memory). The volume of the hippocampus, which is related to memory, is lower in people with elevated blood sugar levels [64,65]. In this study, we considered that hydrogen radicals in natural reduced water may have acted as a reducing agent, similar to vitamin C, and that natural reduced water lowered blood glucose levels, thus improving short-term memory. Natural reduced water activates aquaporins, which are water transport proteins [21]. There is a mechanism for amyloid-β clearance in the brain called the glymphatic system [66], and aquaporin-4 (AQP4) in astrocytes is necessary for this process [67]. Deletion of AQP4 increases amyloid-β accumulation and normal cell atrophy in a mouse model of AD, resulting in memory impairment [68,69]. Single-nucleotide polymorphisms (SNP) in AQP4 are associated with a decline in cognitive measures after the diagnosis of AD [70]. Furthermore, SNP in AQP4 has been associated with Aβ accumulation, disease progression, and cognitive decline [71]. Aquaporin dysfunction may play a role in the accumulation of Aβ and the progression of AD. Therefore, the activation of aquaporins can improve cognitive functions such as short-term memory.

DS-B scores did not improve significantly after the intervention (p = 0.053). However, the improvement in the intervention group and the differences between the groups after the intervention corresponded to a small effect size (Table 3, Table 4). DS-B, such as DS-F, is a test of short-term memory, but is more related to working memory [72]. Prior studies have shown that people with diabetes have working memory deficits [41]. Higher expression levels of heme oxygenase-1 (HO-1), a biomarker of oxidative stress, are associated with lower working memory scores [73]. The oxidative stress marker 8-hydroxy-2′-deoxyguanosine (8-oxoDG) is associated with working memory performance [74]. High levels of LDL cholesterol have also been shown to be associated with poor performance in working memory tasks [75]. Natural reduced water inhibits oxidation and significantly increases HDL cholesterol levels in patients with diabetes [1]. Therefore, the intervention in the current study indirectly affected working memory scores by reducing oxidation or improving blood lipid status.

The percentage of body fat showed a significant improvement before and after the intervention in both groups. Previous studies have shown that water consumption (not natural reduced water) reduces obesity [76,77]. In this study, the body water rate also showed a significant improvement in both groups. Aquaporins regulate body water content [22,23]. In animal cells, natural reduced water-activated aquaporins are significantly more abundant than in tap water; however, in humans, both types of water activate aquaporins to the same extent [21]. This may be because even in the tap water group, drinking water had some effects in this study. Regarding VFT, there was a significant improvement before and after the intervention in the control group alone. The VFT is usually administered orally. However, in this study, it was administered in writing. This may have compromised the reliability of the test results and produced results that were difficult to interpret.

## 5 Limitation

This study has three limitations. First, fluid intake outside the study area may have influenced the study results. As mentioned above, it is possible that the intake of tap water that is not naturally reduced or other ordinary water outside the scope of the study may have some effect on body composition and cognitive function. Furthermore, high fluid intake may be associated with subclinical diseases. Therefore, data on fluid intake and urine output are important because subclinical diseases are difficult to recognize in older adults. However, we were unable to measure these values in this study. In future studies, fluid intake outside of the intervention (total fluid intake) should be recorded. Second, no confounding factors, such as dietary intake, physical activity, alcohol consumption, smoking, and sleeping habits, were collected. Therefore, the effects in this study, uncontrolled by these factors, may be limited to older Japanese adults, as their diets are likely to differ from those of older adults in other parts of the world, and the type and amount of fluid intake may vary from country to country. Although the RCT was controlled for both groups, future studies should focus on the environments of these participants. Finally, this study mainly used cognitive function tests to measure the effect of water intake, but did not perform blood tests. Previous studies have suggested that cognitive function is related to specific biomarkers (blood glucose, ROS, HO-1, GSH, 8-oxoDG, SOD, and aquaporin activity). However, in this study, we did not collect data on these biomarkers because we focused on the cognitive function phenotype in humans as the primary outcome. Future studies should verify these findings by including biomarker measurements.

## 6 Conclusions

This intervention study showed that natural reduced water consumption significantly improved the cognitive functions of attention function and short-term memory in healthy older adults.

## Ethics statement

The Ethics Committee of Tohoku University Graduate School of Medicine (2020-1-631) approved this study.

## Funding statement

The budget for joint research and water was provided by Hita Tenryo-Sui Co., Ltd.

## Additional information

No additional information is available for this paper.

## Data availability statement

The data will be available on request.

## CRediT authorship contribution statement

**Takamitsu Shinada:** Writing – original draft, Resources, Investigation, Formal analysis, Conceptualization. **Keisuke Kokubun:** Writing – review & editing, Formal analysis. **Yuji Takano:** Writing – review & editing, Conceptualization. **Hikari Iki:** Resources, Investigation, Formal analysis, Conceptualization. **Koki Kobayashi:** Resources, Investigation. **Takeki Hamasaki:** Writing – review & editing. **Yasuyuki Taki:** Writing – review & editing, Supervision, Conceptualization.

## Declaration of competing interest

The authors declare the following financial interests/personal relationships which may be considered as potential competing interests:Takamitsu Shinada reports financial support was provided by Hita Tenryo-Sui Co., Ltd.
